# Interstitial pneumonia signals of apalutamide, darolutamide, and enzalutamide in FAERS and JADER

**DOI:** 10.3389/fonc.2026.1889964

**Published:** 2026-08-21

**Authors:** Zihui Zheng, Zinan Zhao, Pengfei Jin

**Affiliations:** Department of Pharmacy, Beijing Hospital, National Center for Gerontology, National Clinical Research Center for Gerontology, The Key Laboratory of Geriatrics of the National Health Commission (NHC), Institute of Geriatric Medicine, Chinese Academy of Medical Sciences, Beijing, China

**Keywords:** apalutamide, darolutamide, enzalutamide, interstitial pneumonia, FAERS, JADER, pharmacovigilance, Weibull analysis

## Abstract

**Background:**

Apalutamide, darolutamide, and enzalutamide are widely used androgen receptor signaling inhibitors (ARSIs) for prostate cancer. Although their clinical benefits are established, interstitial pneumonia remains an uncommon but potentially serious pulmonary adverse event that is insufficiently characterized in routine practice.

**Methods:**

We conducted a retrospective pharmacovigilance study using the FDA Adverse Event Reporting System (FAERS) and the Japanese Adverse Drug Event Report database (JADER). Reports listing apalutamide, darolutamide, or enzalutamide as the primary or suspected drug were identified after drug-name standardization. Interstitial pneumonia/interstitial lung disease was defined at the preferred-term level. Reporting odds ratio (ROR) analysis was used for signal detection; a positive signal was defined as at least three reports and a lower 95% confidence interval of the ROR greater than 1. Time-to-onset patterns were assessed using monthly and cumulative reporting proportions and Weibull distribution analysis.

**Results:**

After data cleaning, 59, 603 target-drug reports were identified in FAERS and 4, 812 in JADER. In FAERS, interstitial lung disease showed positive disproportionality signals for all three ARSIs, with the strongest signal for darolutamide, followed by apalutamide and enzalutamide. In JADER, positive signals were observed for apalutamide and darolutamide, whereas enzalutamide did not meet the predefined signal criterion. Weibull analysis suggested a random-failure pattern for apalutamide in both databases. Darolutamide showed a wear-out pattern in FAERS and a random-failure pattern in JADER. Enzalutamide showed a consistent early-failure pattern in both databases.

**Conclusion:**

The three ARSIs showed heterogeneous interstitial pneumonia reporting patterns in FAERS and JADER. Apalutamide and darolutamide demonstrated more consistent disproportionality signals, whereas enzalutamide showed weaker signal strength but a reproducible early-onset pattern among reports with valid time-to-onset information. These findings support continued pharmacovigilance and early clinical monitoring, while recognizing that spontaneous reporting signals cannot establish causality or true incidence.

## 1 Introduction

Prostate cancer is one of the most common malignancies in men worldwide. GLOBOCAN 2022 estimated approximately 1.47 million new cases and 396, 800 deaths globally, highlighting its substantial disease burden (1). As screening, population aging, and survival after advanced disease continue to increase, long-term systemic treatment has become an increasingly important component of prostate cancer care.

During the past decade, treatment has shifted from androgen-deprivation therapy alone toward intensified regimens incorporating androgen receptor signaling inhibitors (ARSIs). Apalutamide, enzalutamide, and darolutamide have demonstrated clinically meaningful benefits across nonmetastatic castration-resistant, metastatic castration-sensitive, and related high-risk disease states. Pivotal phase III trials showed that these agents delay disease progression and improve clinically relevant outcomes, supporting their widespread use in contemporary practice (2–4).

With broader and earlier use, uncommon but serious adverse events have become more relevant to treatment decisions. Interstitial pneumonia, interstitial lung disease (ILD), and pneumonitis represent overlapping clinical terms for drug-related lung injury that may present with cough, dyspnea, fever, hypoxemia, or ground-glass/interstitial changes on imaging. In oncology patients, attribution is often difficult because infection, tumor progression, radiotherapy-related injury, concomitant drugs, and pre-existing lung disease may produce similar findings. Although ARSI-related ILD appears rare compared with fatigue, rash, hypertension, falls, or central nervous system toxicity, clinically significant cases may lead to treatment interruption, corticosteroid therapy, hospitalization, or death.

Regulatory and post-marketing evidence suggests that pulmonary safety signals may vary by drug and region. In 2019, the Japanese Pharmaceuticals and Medical Devices Agency recommended precaution revisions for apalutamide and enzalutamide related to ILD. Case reports have also described apalutamide-induced ILD in Japanese patients, including cases with dyspnea, bilateral ground-glass or interstitial infiltrates, and improvement after drug withdrawal and corticosteroid treatment (5). By contrast, pulmonary toxicity has received less attention in some darolutamide and enzalutamide labeling materials, leaving the comparative pulmonary safety profile of these agents incompletely defined.

An earlier dual-database pharmacovigilance study evaluated antiandrogen-associated ILD using FAERS and JADER data up to 2019 and found positive signals for bicalutamide and flutamide, but not for enzalutamide or apalutamide (6).

Spontaneous reporting systems are useful for detecting rare or unexpected post-marketing safety signals. FAERS provides a large international source of adverse event reports, while JADER offers structured Japanese post-marketing data and is particularly relevant for drug-induced lung injury in Japan. These databases are complementary, but their results must be interpreted as reporting disproportionality signals rather than causal effects or population incidence because they lack complete exposure denominators and are affected by underreporting, stimulated reporting, missing information, and duplicate reports.

We therefore conducted a dual-database pharmacovigilance study using FAERS and JADER to characterize interstitial pneumonia/interstitial lung disease signals associated with apalutamide, darolutamide, and enzalutamide. We compared respiratory preferred-term profiles across databases and evaluated time-to-onset patterns using monthly/cumulative reporting proportions and Weibull distribution analysis. The aim was to provide updated real-world evidence to support early recognition and risk management of this uncommon but clinically important pulmonary event during ARSI therapy.

## 2 Materials and methods

### 2.1 Study design and data sources

This retrospective pharmacovigilance study used two spontaneous reporting systems: FAERS and JADER. The analysis focused on interstitial pneumonia associated with apalutamide, darolutamide, and enzalutamide.

For FAERS, the DEMO, DRUG, REAC, OUTC, INDI, THER, and PRSR files were extracted and linked. For JADER, the corresponding demographic, drug, adverse event, and related tables were integrated using unique report identifiers. After cleaning and deduplication, the processed FAERS dataset included 19, 906, 480 demographic records, 72, 576, 005 drug records, and 59, 176, 187 adverse event records. The processed JADER dataset included 996, 199 reports, 4, 839, 435 drug records, and 1, 650, 208 adverse event records.

FAERS and JADER are publicly available anonymized pharmacovigilance databases; therefore, ethical approval and informed consent were not required.

### 2.2 Data processing

Data processing was performed separately for the two databases. In FAERS, duplicate reports were removed by retaining the latest and most complete version of each case according to report identifiers and follow-up information. The cleaned DEMO, DRUG, REAC, OUTC, INDI, THER, and PRSR tables were then linked by report identifier.

In JADER, demographic, drug, and adverse event records were linked using unique case identifiers. Drug records were restricted to suspected drugs (被疑薬). In FAERS, only reports in which the target drug was recorded as the primary suspect drug were included (role_cod = PS). Reports with missing drug names, missing adverse event terms, invalid linkage information, or non-target drug roles were excluded from the corresponding analyses.

### 2.3 Drug identification

The target drugs were apalutamide, darolutamide, and enzalutamide. Drug names were standardized using generic names, brand names, development codes, and Japanese product names when applicable. FAERS matching included English generic and brand names, such as apalutamide/Erleada, darolutamide/Nubeqa, and enzalutamide/Xtandi. JADER matching used both Japanese and English generic and product names.

### 2.4 Definition of interstitial pneumonia

The adverse event of interest was interstitial pneumonia. The primary endpoint was defined using the corresponding MedDRA preferred term related to interstitial pneumonia or interstitial lung disease. Japanese adverse event terms in JADER were harmonized to the same clinical concept when applicable.For the purposes of this analysis, the terms “interstitial pneumonia” and “interstitial lung disease” refer to the same prespecified pharmacovigilance endpoint, reflecting database-specific MedDRA terminology and clinical usage.

To maintain a focused endpoint, non-specific respiratory events and other pulmonary events, including cough, dyspnea, pulmonary embolism, pleural effusion, pulmonary oedema, and pulmonary toxicity, were not included in the primary endpoint. Pulmonary fibrosis was not treated as an independent primary endpoint unless it was coded within the interstitial pneumonia/interstitial lung disease definition used for the main analysis.

### 2.5 Disproportionality analysis

Signal detection was performed using the reporting odds ratio (ROR). FAERS and JADER were analyzed separately to evaluate whether interstitial pneumonia was disproportionately reported with apalutamide, darolutamide, or enzalutamide compared with all other drugs in the same database.

A positive signal was defined as at least three reports for the drug-event pair and a lower limit of the 95% confidence interval of the ROR greater than 1.0. ROR values and 95% confidence intervals were calculated for each target drug and database. Because spontaneous reporting systems lack complete exposure denominators and are subject to reporting bias, signal results were interpreted as reporting disproportionality rather than incidence or causality (7).

### 2.6 Descriptive analysis

Reports involving both a target ARSI and interstitial pneumonia were summarized separately for FAERS and JADER. Available variables included age, sex, reporting year, reporter type, serious outcomes, hospitalization, life-threatening events, and death. Continuous variables were described using medians and interquartile ranges; categorical variables were summarized as counts and percentages.

### 2.7 Time-to-onset analysis

Time to onset was calculated among interstitial pneumonia reports with valid date information as the interval in days between the recorded start date of the target ARSI and the recorded onset date or event-related date of interstitial pneumonia.

Reports were excluded if the drug start date or event date was missing, incomplete, inconsistent, or generated a non-positive interval. For JADER records containing multiple date entries in a single field, date strings were split and the first complete valid start-event date pair was used. Year-only or year-month-only dates were not imputed.

Time-to-onset values were grouped into 30-day intervals from 0–1 month to 17–18 months; events after 18 months were classified as >18 months. Monthly and cumulative reporting proportions were calculated among reports with valid time-to-onset information. These proportions describe onset distribution among analyzable reports and do not represent population-based incidence.

### 2.8 Weibull distribution analysis

Weibull distribution analysis was used to characterize the temporal pattern of interstitial pneumonia after ARSI initiation. The Weibull probability density function was fitted to observed time-to-onset data by maximum likelihood estimation. The scale parameter α and shape parameter β were estimated for each drug and database.

Bootstrap resampling was used to estimate 95% confidence intervals for α and β. Failure type was classified according to β: an upper 95% confidence limit below 1 indicated an early-failure pattern; a 95% confidence interval including 1 indicated a random-failure pattern; and a lower 95% confidence limit above 1 indicated a wear-out pattern (8).

Histograms of observed onset times were overlaid with fitted Weibull curves. Each plot displayed the number of analyzable reports, median onset time, interquartile range, minimum-to-maximum range, α, β, 95% confidence intervals, and failure type.

### 2.9 Statistical analysis

FAERS and JADER were analyzed independently and then compared descriptively to assess consistency of reporting patterns. No direct comparison of true incidence was performed because neither database provides complete information on exposed populations.

All analyses were performed using Python and Microsoft Excel. Data cleaning, aggregation, signal calculation, time-to-onset processing, Weibull fitting, bootstrap estimation, and figure generation were conducted using Python with pandas, numpy, scipy, and matplotlib. Microsoft Excel was used for data verification, table organization, and manual cross-checking of key outputs. High-resolution figures were exported for manuscript preparation.

## 3 Results

### 3.1 Data extraction and baseline characteristics

**Figure 1:**
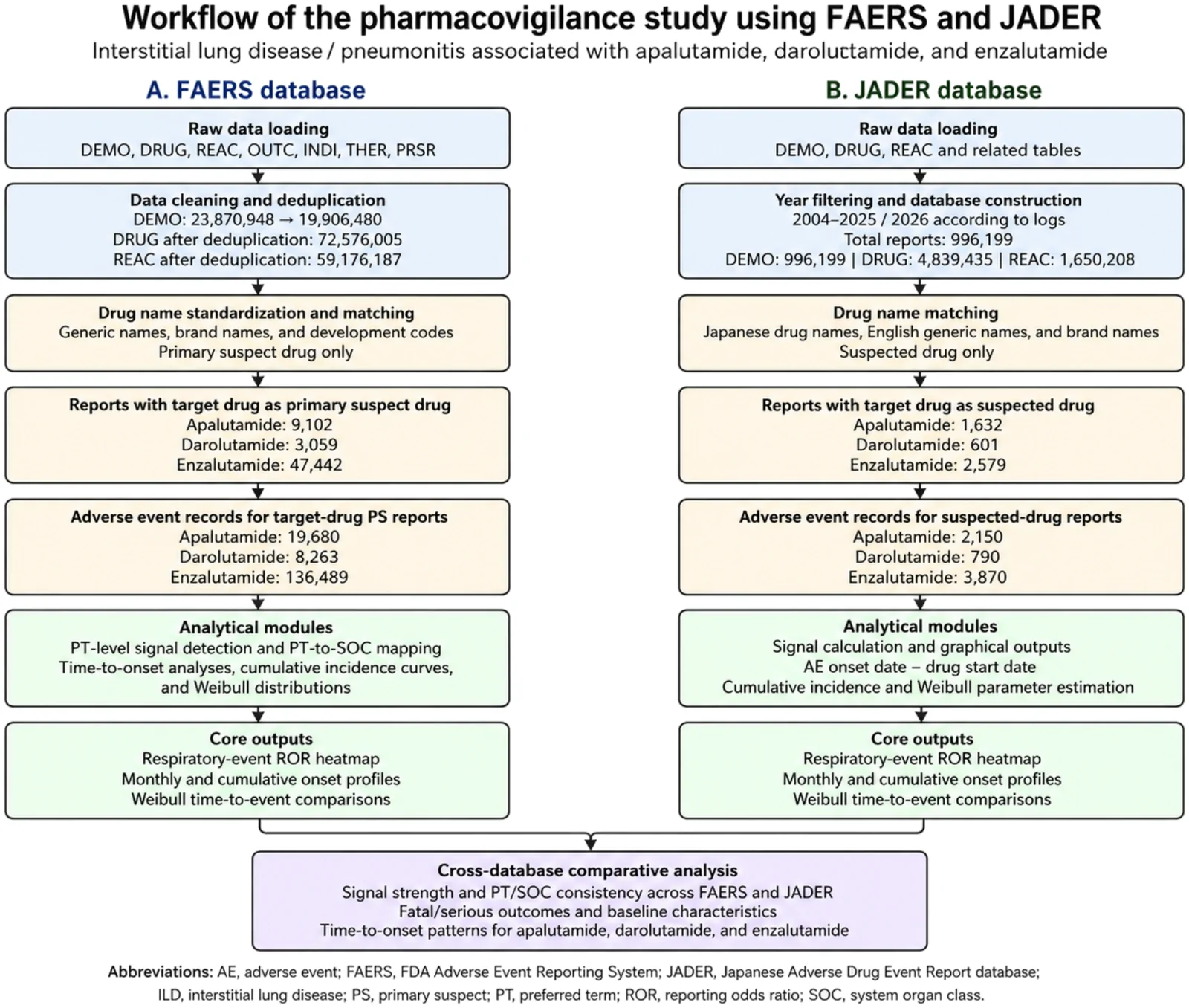
Flow diagram of data extraction and analysis in FAERS and JADER. **(A)** FAERS data; **(B)** JADER data.

The overall workflow for data extraction, cleaning, drug identification, and interstitial pneumonia analysis is shown in **Figure 1**. After deduplication and database integration, the analytic FAERS dataset contained 19, 906, 480 demographic records, 72, 576, 005 drug records, and 59, 176, 187 adverse event records. The processed JADER dataset contained 996, 199 reports, 4, 839, 435 drug records, and 1, 650, 208 adverse event records.

After drug-name standardization and restriction to primary/suspected drug reports, 59, 603 target-drug reports were identified in FAERS: 9, 102 for apalutamide, 3, 059 for darolutamide, and 47, 442 for enzalutamide. These reports corresponded to 164, 432 adverse event records. In JADER, 4, 812 target-drug reports were identified: 1, 632 for apalutamide, 601 for darolutamide, and 2, 579 for enzalutamide, corresponding to 6, 810 adverse event records.

Enzalutamide accounted for the largest proportion of target-drug reports in both databases. Most reports involved male patients, consistent with the approved indications of these agents. Compared with FAERS, JADER reports were more concentrated in older patients, particularly those aged 75–89 years. Reporter sources also differed: consumer reports contributed substantially to FAERS, whereas physician reports were more prominent in JADER. Baseline characteristics are summarized in **Table 1**.

**Table 1:** Baseline characteristics of target-drug reports in FAERS and JADER.

Characteristic	FAERS			JADER		
Characteristic	Apalutamide	Darolutamide	Enzalutamide	Apalutamide	Darolutamide	Enzalutamide
Overall						
Target-drug reports, n	9, 102	3, 059	47, 442	1, 632	601	2, 579
Target-drug adverse event records, n	19, 680	8, 263	136, 489	2, 150	790	3, 870
Reporting period; peak year, n (%)	2018–2025; peak 2022: 1, 810 (19.9)	2019–2025; peak 2025: 1, 024 (33.5)	2012–2025; peak 2017: 9, 642 (20.3)	2015–2025; peak 2020: 278 (17.0)	2020–2025; peak 2024: 254 (42.3)	2014–2025; peak 2014: 385 (14.9)
Demographic characteristics						
Male	8, 117 (89.2)	2, 964 (96.9)	46, 505 (98.0)	1, 631 (99.9)	587 (97.7)	2, 565 (99.5)
Female	36 (0.4)	14 (0.5)	271 (0.6)	1 (0.1)	2 (0.3)	0 (0.0)
Missing sex	949 (10.4)	81 (2.6)	666 (1.4)	0 (0.0)	12 (2.0)	14 (0.5)
<60 years	185 (2.0)	172 (5.6)	1, 515 (3.2)	28 (1.7)	16 (2.7)	35 (1.4)
60–74 years	1, 844 (20.3)	898 (29.4)	10, 173 (21.4)	138 (8.5)	91 (15.1)	225 (8.7)
75–89 years	2, 505 (27.5)	878 (28.7)	13, 858 (29.2)	1, 096 (67.2)	270 (44.9)	1, 564 (60.6)
≥90 years	436 (4.8)	137 (4.5)	2, 092 (4.4)	120(7.4)	20 (3.3)	263 (10.2)
Missing age	4, 132 (45.4)	974 (31.8)	19, 804 (41.7)	250 (15.3)	204 (33.9)	492 (19.1)
Report characteristics						
physician	2, 455 (27.0)	938 (30.7)	6, 142 (12.9)	1, 273 (78.0)	155 (25.8)	746 (28.9)
pharmacist	814 (8.9)	157 (5.1)	2, 196 (4.6)	157 (9.6)	36 (6.0)	246 (9.5)
other healthcare professional	1, 727 (19.0)	484 (15.8)	619 (1.3)	145 (8.9)	53 (8.8)	52 (2.0)
consumer	3, 195 (35.1)	1, 457 (47.6)	33, 354 (70.3)	7 (0.4)	6 (1.0)	97 (3.8)
other	911 (10.0)	23 (0.8)	5, 131 (10.8)	50 (3.1)	351 (58.4)	1, 438 (55.8)

AE, adverse event; FAERS, FDA Adverse Event Reporting System; JADER, Japanese Adverse Drug Event Report database.

### 3.2 Respiratory SOC signal profile

**Figure 2:**
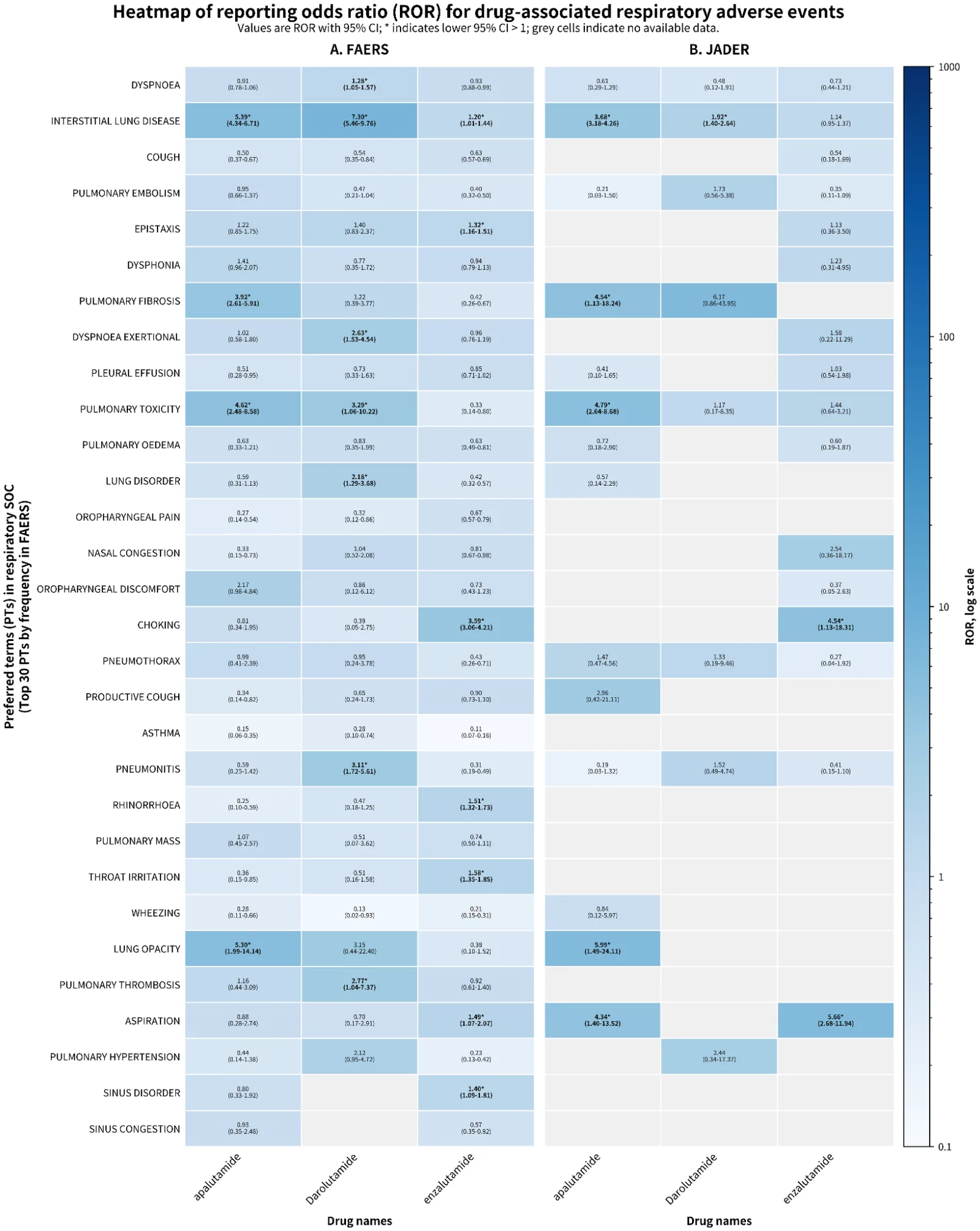
Respiratory SOC signal profile of apalutamide, darolutamide, and enzalutamide in FAERS and JADER. **(A)** FAERS data; **(B)** JADER data.

The respiratory SOC signal profile is shown in **Figure 2**. In FAERS, interstitial lung disease showed positive disproportionality signals for all three ARSIs, with the strongest signal for darolutamide (ROR 7.30, 95% CI 5.46-9.76), followed by apalutamide (ROR 5.39, 95% CI 4.34-6.71) and enzalutamide (ROR 1.20, 95% CI 1.01-1.44). In JADER, positive signals were detected for apalutamide (ROR 3.68, 95% CI 3.18-4.26) and darolutamide (ROR 1.92, 95% CI 1.40-2.64), whereas enzalutamide did not meet the predefined signal criterion.

Other pulmonary preferred terms showed more heterogeneous patterns. Pulmonary toxicity was positively associated with apalutamide and darolutamide in FAERS and with apalutamide in JADER. Pulmonary fibrosis showed a positive signal for apalutamide in both databases, while darolutamide had a numerically increased but imprecise estimate in JADER. Pneumonitis showed a positive signal only for darolutamide in FAERS.

Overall, the most consistent and clinically relevant signal was concentrated in interstitial lung disease, particularly for apalutamide and darolutamide across the two databases. This pattern supported focusing subsequent analyses on interstitial pneumonia/interstitial lung disease rather than broader non-specific respiratory events.

### 3.3 Time-to-onset pattern and Weibull distribution analysis

**Figure 3:**
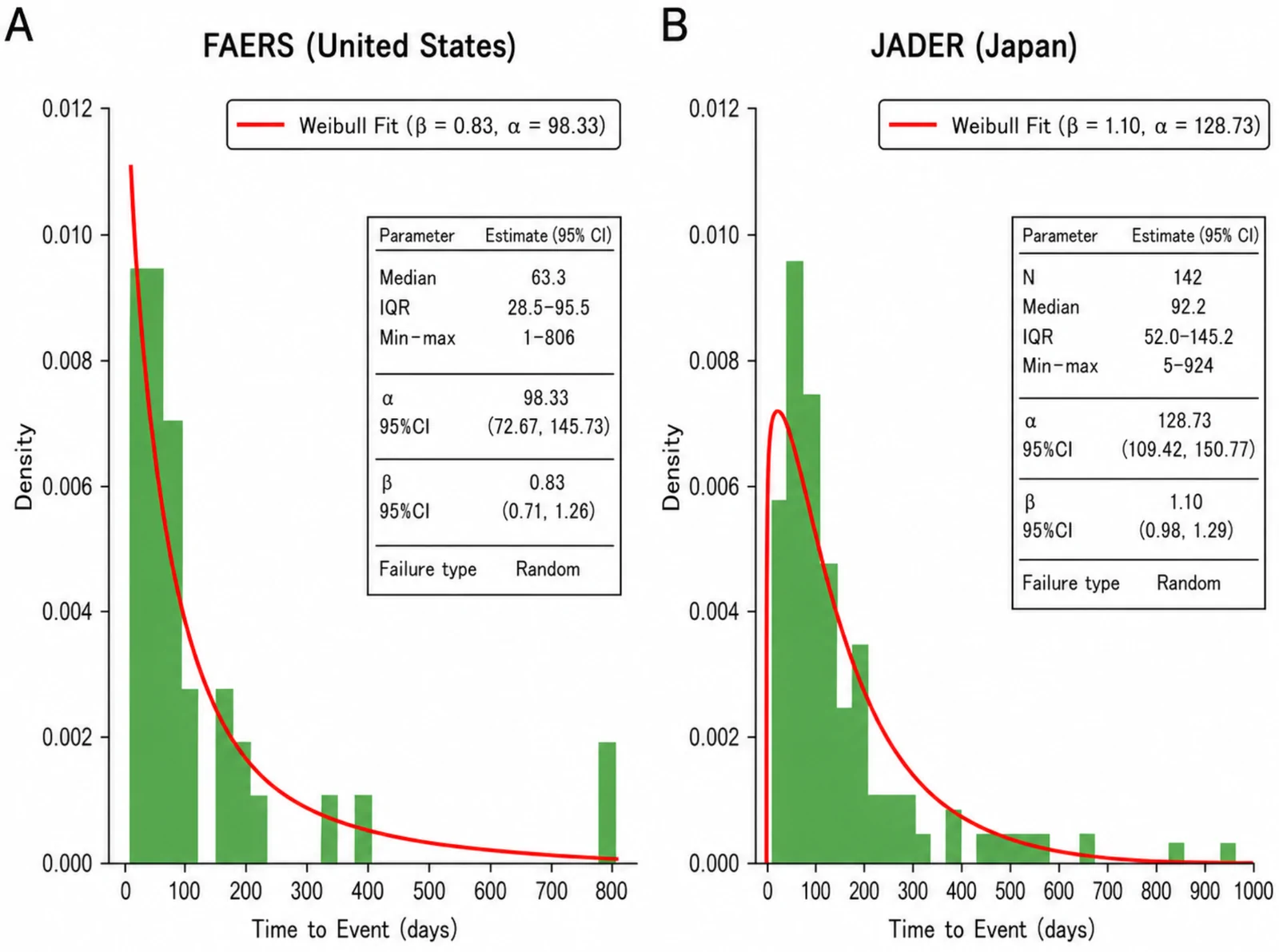
Weibull distribution analysis of apalutamide-associated interstitial pneumonia in FAERS and JADER. The Weibull shape parameter β and its 95% confidence interval were used to classify the time-to-onset pattern as early failure, random failure, or wear-out relative to 1. **(A)** FAERS analysis; **(B)** JADER analysis.

**Figure 4:**
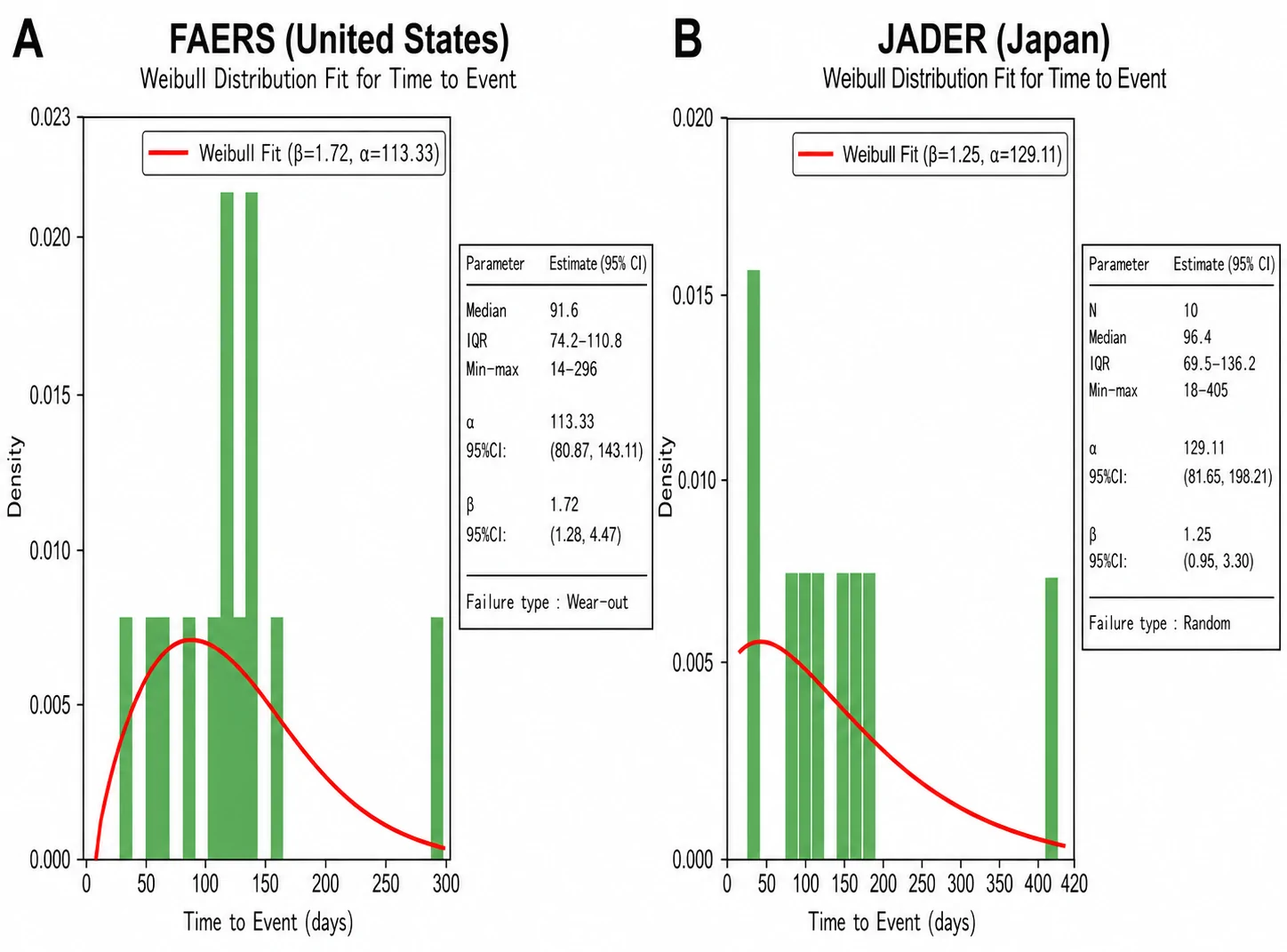
Weibull distribution analysis of darolutamide-associated interstitial pneumonia in FAERS and JADER. The Weibull shape parameter β and its 95% confidence interval were used to classify the time-to-onset pattern as early failure, random failure, or wear-out relative to 1. **(A)** FAERS data; **(B)** JADER data.

**Figure 5:**
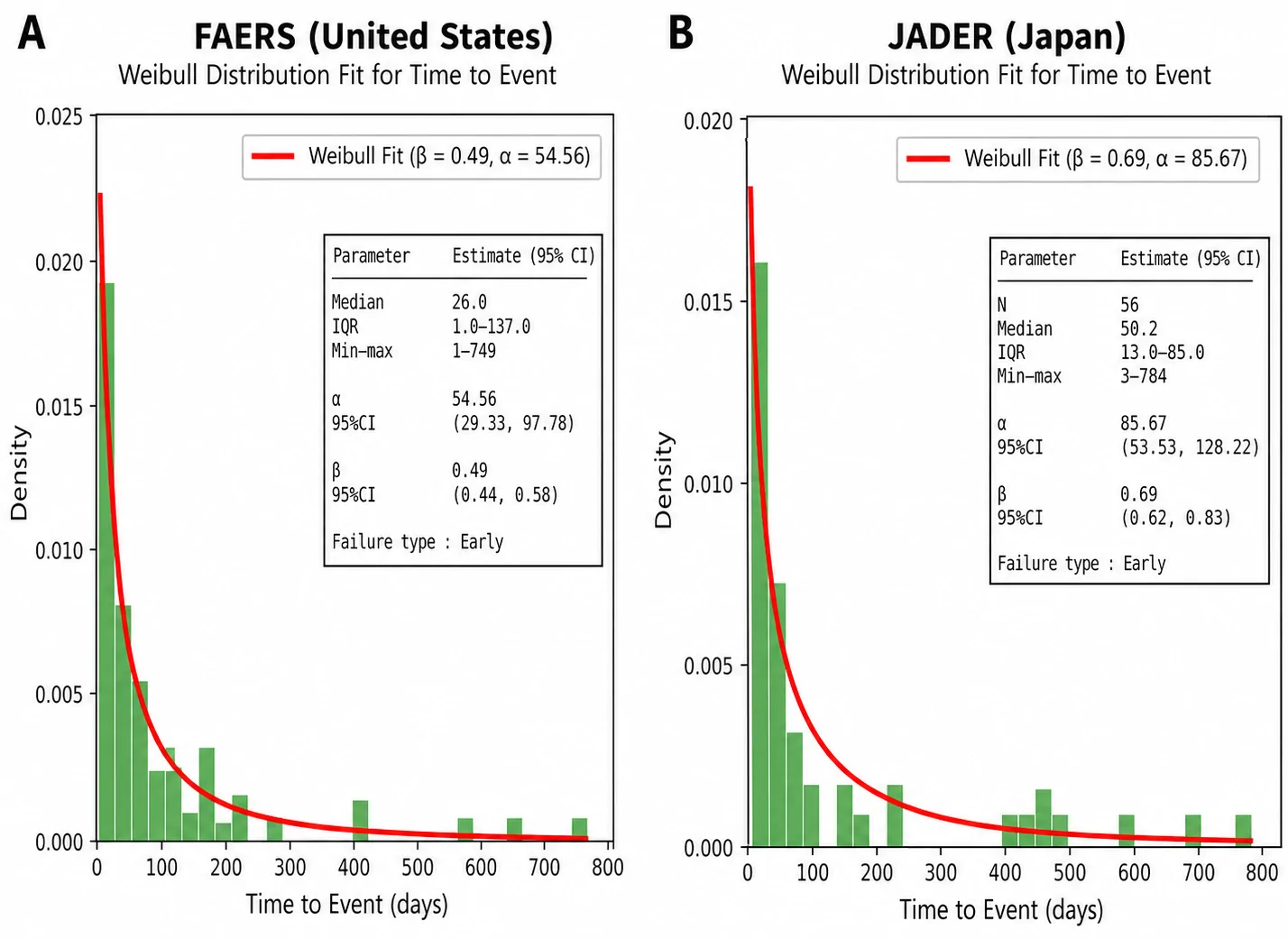
Weibull distribution analysis of enzalutamide-associated interstitial pneumonia in FAERS and JADER. The Weibull shape parameter β and its 95% confidence interval were used to classify the time-to-onset pattern as early failure, random failure, or wear-out relative to 1. **(A)** FAERS data; **(B)** JADER data.

Weibull distribution analysis was used to characterize reported time-to-onset patterns of interstitial pneumonia for each ARSI in FAERS and JADER (**Figures 3**–**5**). The temporal patterns differed by drug, with partial consistency across databases.

For apalutamide, the Weibull shape parameter suggested a random-failure pattern in both databases. In FAERS, the median onset time was 63.3 days (IQR, 28.5-95.5), with α = 98.33 and β = 0.83 (95% CI, 0.71-1.26). In JADER, the median onset time was 92.2 days (IQR, 52.0-145.2), with α = 128.73 and β = 1.10 (95% CI, 0.98-1.29). Because the β confidence intervals included 1, apalutamide was not classified as clearly early-onset or delayed-onset (**Figure 3**).

Darolutamide showed different patterns between databases. In FAERS, the median onset time was 91.6 days (IQR, 74.2-110.8), with α = 113.33 and β = 1.72 (95% CI, 1.28-4.47), indicating a wear-out pattern. In JADER, the median onset time was 96.4 days (IQR, 69.5-136.2), with α = 129.11 and β = 1.25 (95% CI, 0.95-3.30), corresponding to a random-failure pattern. Because only 10 JADER reports were analyzable, this discrepancy should be interpreted cautiously (**Figure 4**).

Enzalutamide showed a consistent early-failure pattern in both databases. In FAERS, the median onset time was 26.0 days (IQR, 1.0-137.0), with α = 54.56 and β = 0.49 (95% CI, 0.44-0.58). In JADER, after harmonizing the time-to-onset dataset to 56 reports, the median onset time was 50.2 days (IQR, 13.0-85.0), with α = 85.67 and β = 0.69 (95% CI, 0.62-0.83). In both databases, the upper confidence limit for β was below 1, supporting an early-onset profile (**Figure 5**).

Taken together, the reported onset pattern of interstitial pneumonia was drug-specific. Enzalutamide showed the most consistent early-onset profile, apalutamide showed a random pattern, and darolutamide showed a possible delayed pattern in FAERS but not in JADER. These findings support close monitoring after ARSI initiation while recognizing that later-onset events may also occur.

### 3.4 Monthly and cumulative time-to-onset profiles

**Figure 6:**
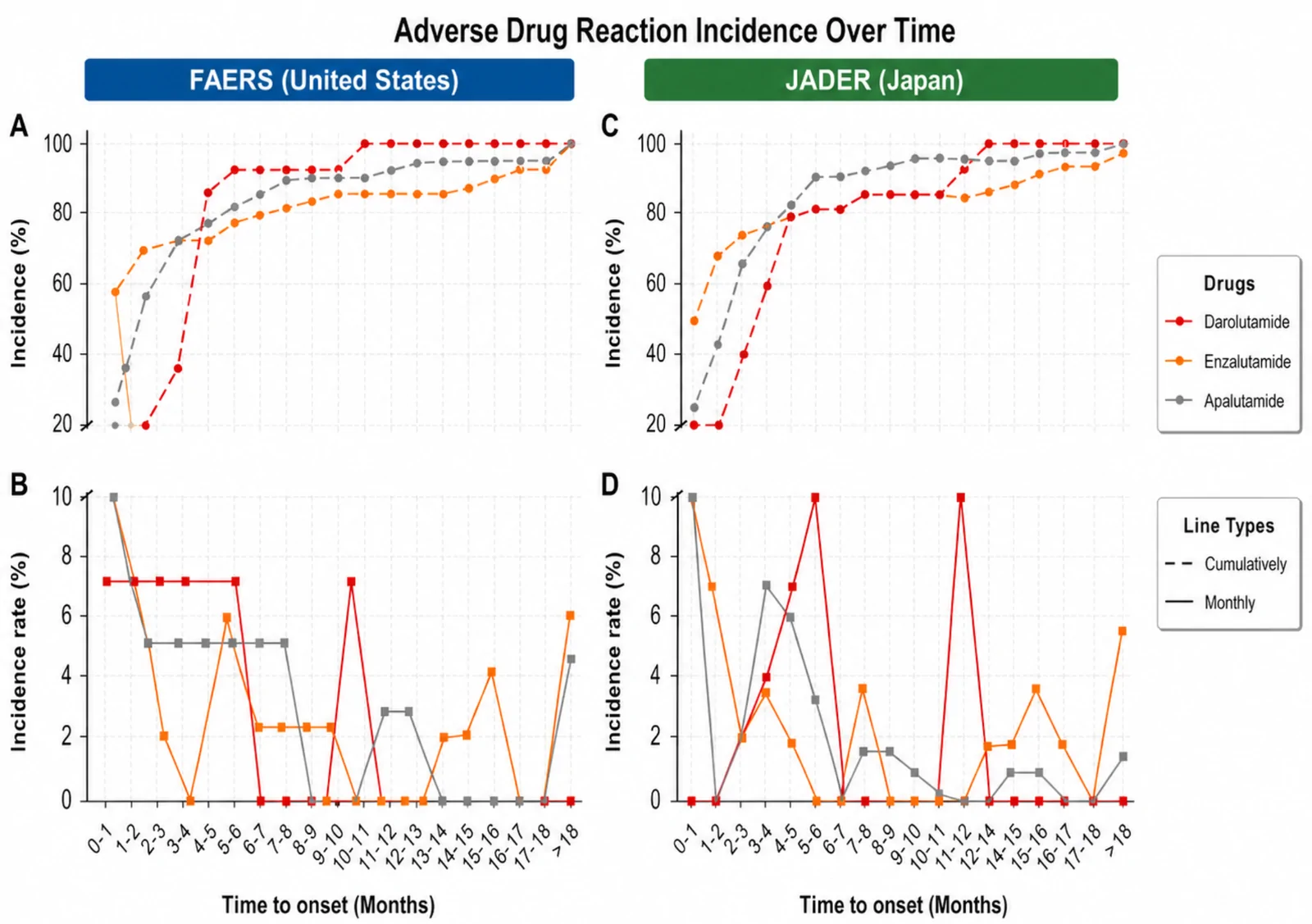
Multi-panel plot of monthly and cumulative time-to-onset profiles of interstitial pneumonia associated with apalutamide, darolutamide, and enzalutamide in FAERS and JADER. **(A, B)** FAERS data; **(C, D)** JADER data.

Monthly and cumulative time-to-onset profiles are shown in **Figure 6**. The plotted values represent the proportion of reports among cases with valid time-to-onset information, not population-based incidence rates.

In **Figure 6A**, cumulative reporting proportions in FAERS differed across drugs. Enzalutamide accumulated most rapidly, with more than half of analyzable reports occurring within the first month and approximately 70% within the first 2 months. Apalutamide increased more gradually over the first several months. Darolutamide showed a stepwise rise, with a marked increase around months 3–5 and cumulative capture by approximately 10 months.

**Figure 6B** shows the corresponding monthly reporting proportions in FAERS. Enzalutamide had a prominent first-month peak followed by smaller contributions across later intervals. Apalutamide showed several early-to-intermediate monthly contributions rather than a single dominant peak. Darolutamide showed intermittent peaks, consistent with a less continuous onset distribution.

In **Figure 6C**, JADER cumulative curves also showed early accumulation but with different relative patterns. Enzalutamide again reached approximately half of reports within the first month. Apalutamide increased sharply during the first 3–4 months and then approached a plateau. Darolutamide showed a stepwise cumulative pattern, but the curve was unstable because of the small number of analyzable reports.

**Figure 6D** shows discrete monthly peaks in JADER rather than smooth distributions. Enzalutamide had the largest earliest monthly contribution and smaller intermittent peaks thereafter. Apalutamide showed early monthly peaks consistent with the cumulative pattern. Darolutamide showed isolated peaks at specific intervals, which should be interpreted cautiously given the limited sample size.

Overall, **Figure 6** indicates that many reports occurred during the early treatment period, particularly for enzalutamide in both databases. Apalutamide showed early-to-intermediate accumulation, while darolutamide displayed more database-dependent and stepwise patterns. Continued vigilance remains necessary because late-onset reports were also observed.

## 4 Discussion

This dual-database pharmacovigilance study identified heterogeneous interstitial pneumonia reporting patterns for apalutamide, darolutamide, and enzalutamide. The respiratory SOC-related profile suggested that pulmonary reporting signals differed across the three ARSIs. Interstitial lung disease was the most consistent clinically relevant signal, particularly for apalutamide and darolutamide. Enzalutamide showed weaker disproportionality, especially in JADER, but had a reproducible early-onset pattern among reports with valid time-to-onset information. These findings argue against treating ARSI-associated interstitial pneumonia as a uniform class effect; rather, the signal appears drug-specific and database-dependent.

The broader respiratory PT comparison helps place the interstitial pneumonia results in context. In FAERS, all three drugs met the predefined signal criterion for interstitial lung disease, with the strongest signal for darolutamide. In JADER, only apalutamide and darolutamide met the criterion. Apalutamide showed the most consistent cross-database pulmonary profile, including signals for pulmonary fibrosis and pulmonary toxicity. Darolutamide had a strong FAERS-based signal but a less stable JADER profile. Enzalutamide had the weakest respiratory disproportionality profile but a clearer early temporal pattern. Thus, the pulmonary signal profile differed not only by drug but also by the analytic dimension considered.

An earlier dual-database analysis of FAERS and JADER data up to 2019 found positive ILD signals for bicalutamide and flutamide, but not for enzalutamide or apalutamide (6). At that time, post-marketing exposure to newer ARSIs was limited, particularly for apalutamide and darolutamide. The positive signals observed in the present study may reflect expanded use, longer follow-up, increased clinical recognition of pulmonary toxicity, or differences in reporting accumulation. They should not be interpreted as evidence that the underlying biological risk has increased.

The apalutamide findings are consistent with current regulatory and clinical evidence. U.S. prescribing information states that fatal and life-threatening ILD/pneumonitis can occur with apalutamide and recommends monitoring for dyspnea, cough, and fever, withholding treatment when ILD/pneumonitis is suspected, and permanently discontinuing treatment in severe cases or when no other cause is identified (9). Japanese case reports also support clinical plausibility, describing dyspnea, interstitial infiltrates or ground-glass opacities, and improvement after apalutamide withdrawal and corticosteroid therapy (5, 10, 11). In our analysis, apalutamide showed positive interstitial lung disease signals in both databases, but the Weibull pattern was random rather than clearly early-onset. This suggests a relatively broad onset window.

The darolutamide signal requires particular caution. Darolutamide showed the strongest FAERS signal for interstitial lung disease and also met the JADER signal criterion. This is noteworthy because darolutamide is often considered comparatively well tolerated, especially with respect to central nervous system events. However, our findings do not establish a higher true incidence of interstitial pneumonia with darolutamide. The FAERS wear-out pattern and the JADER random pattern may reflect small numbers, incomplete date information, shorter post-marketing experience, or database-specific reporting practices rather than a true pharmacological difference. The JADER darolutamide time-to-onset analysis included only 10 reports, so temporal conclusions should remain conservative.

For enzalutamide, the signal was weaker but temporally consistent. FAERS showed a modest positive signal for interstitial lung disease, while JADER did not meet the signal criterion. Nevertheless, enzalutamide showed an early-failure pattern in both databases among reports with valid date information. This indicates that when enzalutamide-associated interstitial pneumonia is reported, onset tends to cluster early after treatment initiation. The negative JADER disproportionality result should therefore not be read as absence of risk; it may reflect lower relative reporting frequency, coding differences, competing background reports, or other database-specific factors.

The time-to-onset analyses add clinically relevant information beyond signal strength. Enzalutamide reports accumulated rapidly within the first 1–2 months in both databases, apalutamide showed early-to-intermediate accumulation, and darolutamide showed a more stepwise pattern. Early clustering has also been described for other targeted therapy-related ILD, including updated FAERS analyses of ALK inhibitors, although the drug class and cancer setting are different. Our findings therefore support heightened attention during the first several months after ARSI initiation, while avoiding the assumption that later-onset events are unlikely.

Differences between FAERS and JADER are central to interpretation. FAERS contains a larger and more heterogeneous reporting population, including reports from healthcare professionals, manufacturers, and consumers. JADER reflects Japanese post-marketing reporting and may be influenced by local prescribing patterns, regulatory communication, coding conventions, and awareness of drug-induced lung injury. In our dataset, FAERS included many more target-drug reports, whereas JADER reports were more concentrated in older patients and had different reporter-source distributions. Spontaneous reporting data cannot determine whether these differences reflect biological susceptibility, exposure patterns, reporting behavior, or regulatory attention.

Recent broader pharmacovigilance work also supports drug-specific evaluation among androgen receptor antagonists. A 2025 WHO-VigiAccess analysis reported distinct adverse reaction profiles for apalutamide, darolutamide, and enzalutamide (10). That study was mainly descriptive and did not focus on interstitial pneumonia or dual-database time-to-onset patterns. Our analysis complements this evidence by focusing on a clinically serious pulmonary endpoint and by integrating ROR-based signal detection with Weibull and cumulative onset analyses.

Clinically, these findings support vigilance rather than avoidance. ARSIs provide substantial disease-control and survival benefits in prostate cancer, and pharmacovigilance signals alone do not justify withholding these agents from appropriate patients. However, new cough, dyspnea, fever, hypoxemia, or interstitial changes on imaging during ARSI therapy should prompt evaluation for interstitial pneumonia alongside alternative causes such as infection, tumor progression, radiotherapy injury, pulmonary embolism, heart failure, and concomitant medications. For apalutamide, labeling already provides specific management guidance. For darolutamide and enzalutamide, our results support continued post-marketing monitoring and individualized clinical assessment rather than definitive causal conclusions.

This study has limitations. FAERS and JADER are spontaneous reporting databases and are subject to underreporting, duplicate reporting, stimulated reporting, missing information, and reporting bias. Neither database provides complete exposure denominators, so ROR values and reporting proportions cannot be interpreted as incidence rates. Interstitial pneumonia was identified through reported adverse event terms rather than adjudicated diagnoses, and information on imaging, laboratory tests, baseline lung disease, smoking history, radiotherapy, infection workup, and concomitant drugs was incomplete. Time-to-onset analysis was restricted to reports with valid dates, which may introduce selection bias. Some estimates, particularly darolutamide in JADER, were based on small numbers. Finally, disproportionality analysis can identify signals but cannot establish causality.

The study also has strengths. It provides an updated dual-database evaluation of interstitial pneumonia for three widely used ARSIs, applies consistent drug-role restrictions and endpoint definitions, and combines disproportionality analysis with temporal characterization. The FAERS-JADER comparison offers complementary information on global and Japanese reporting patterns, while the Weibull and monthly/cumulative analyses help define when reported events tend to occur.

In summary, apalutamide showed the most consistent cross-database pulmonary signal profile, darolutamide showed the strongest FAERS disproportionality signal with a more uncertain temporal pattern, and enzalutamide showed weaker database-level disproportionality but reproducible early onset among analyzable reports. These findings support continued pharmacovigilance and early clinical monitoring for interstitial pneumonia during ARSI therapy, while emphasizing that spontaneous reporting signals remain hypothesis-generating.

## 5 Conclusion

Apalutamide, darolutamide, and enzalutamide showed heterogeneous interstitial pneumonia reporting patterns in FAERS and JADER. Apalutamide and darolutamide demonstrated more consistent disproportionality signals across databases, whereas enzalutamide showed weaker signal strength but a reproducible early-onset pattern among reports with valid time-to-onset information. Interstitial pneumonia should therefore be considered an uncommon but clinically relevant pulmonary safety concern during ARSI therapy, particularly during the first several months after initiation. Because spontaneous reporting data cannot establish causality or true incidence, further clinical and epidemiological studies are needed.

## Data Availability

The original contributions presented in the study are included in the article/supplementary material. Further inquiries can be directed to the corresponding author.
